# Lattice Structures—Mechanical Description with Respect to Additive Manufacturing

**DOI:** 10.3390/ma17215298

**Published:** 2024-10-31

**Authors:** Karel Ráž, Zdeněk Chval, Mathis Pereira

**Affiliations:** 1 Faculty of Mechanical Engineering, Regional Technological Institute, University of West Bohemia, Univerzitni 2732/8, 301 00 Plzen, Czech Republic; zdchval@fst.zcu.cz; 2 Polytech Montpellier, Selective Engineering School, Pl. Eugene Bataillon, 34090 Montpellier, France; mathis.pereira-de-pinho@etu.umontpellier.fr

**Keywords:** additive manufacturing, lattice structures, compression, MJF

## Abstract

Lattice structures, characterized by their repetitive, interlocking patterns, provide an efficient balance of strength, flexibility, and reduced weight, making them essential in fields such as aerospace and automotive engineering. These structures use minimal material while effectively distributing stress, providing high resilience, energy absorption, and impact resistance. Composed of unit cells, lattice structures are highly customizable, from simple 2D honeycomb designs to complex 3D TPMS forms, and they adapt well to additive manufacturing, which minimizes material waste and production costs. In compression tests, lattice structures maintain stiffness even when filled with powder, suggesting minimal effect from the filler material. This paper shows the principles of creating finite element simulations with 3D-printed specimens and with usage of the lattice structure. The comparing of simulation and real testing is also shown in this research. The efficiency in material and energy use underscores the ecological and economic benefits of lattice-based designs, positioning them as a sustainable choice across multiple industries. This research analyzes three selected structures—solid material, pure latices structure, and boxed lattice structure with internal powder. The experimental findings reveal that the simulation error is less than 8% compared to the real measurement. This error is caused by the simplified material model, which is considering the isotropic behavior of the used material PA12GB (not the anisotropic model). The used and analyzed production method was multi jet fusion.

## 1. Introduction

Lattice structures are essential across numerous fields, including materials science and engineering, due to their exceptional balance of strength, flexibility, and low weight [1,2]. Their repetitive, interlocking patterns help distribute stress evenly throughout the structure, making them highly efficient in load-bearing applications while minimizing material use [3,4]. This makes them especially valuable in industries such as aerospace, automotive, and construction, where reducing weight without sacrificing strength is critical. Lattice structures also possess high resilience, allowing them to effectively absorb and dissipate energy, which is advantageous for impact resistance and shock absorption [5]. Beyond their mechanical benefits, they offer thermal and acoustic insulation and have large ratio between surface and volume, which is making them useful in chemical reactions and filtration systems. Their versatility and efficiency position lattice structures as key components in driving sustainable, high-performance designs across various industries [6,7,8]. However, there is currently no widely adopted approach for designing processes that incorporate 3D-printed structures (not limited to lattices). Accurately describing the mechanical properties of these structures is essential for their correct application in different contexts [9,10]. Additionally, it is important to properly outline the design process to maximize the productivity of additive manufacturing (AM) technologies. In the compression test conducted, testing bricks were used, and mechanical testing was compared with virtual simulation results. There are also specialized AM techniques such as multi jet fusion (MJF), which allows non-melted powder to remain in the internal cavities of the product. However, there is no established method for managing this residual powder at present.

## 2. What Are Lattice Structures?

Lattice structures are architected materials with complex repetitive, periodic, nonperiodic, or even stochastic patterns. By placing material in specific locations and arranging structures, lattice structures allow the creation of a larger structure with inimitable mechanical properties. Those structures can take many shapes, such as strut-based lattices, which, as scaffolding, are made of rods oriented in different directions, distributing efforts in the structure. But lattice can take much more intricate shapes, such as triply periodic minimal surface (TPMS) structures [11].

Despite the diversity of their geometries, lattice structures have two things in common: a significant reduction in the density of the structures they make up and extreme difficulty in machining [12,13]. The latter makes lattice structures a great candidate for 3D printing/additive manufacturing (AM). In fact, by drastically diminishing the mass of each part, the printing time and material usage of such structures also diminishes subsequently in most cases [14,15,16].

**Figure 1:**
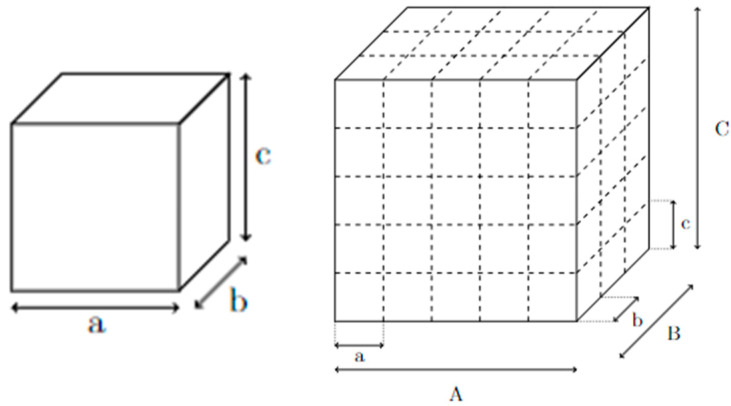
Unit cell of lattice structure (**left**) and complex structure (**right**) with parameters.

Lattice structures are generally the repetition of a unit cell (Figure 1). The unit cell is a basic building block of all structures made of lattice. Each unit cell can be described as a building block for the general structure [17]. Most of the time, it is represented by its length, width, height, and the thickness of the elements composing the unit cell [18]. In Figure 1, a, b, and c are the dimensions of the unit cell, and n_a_ = A/a is the number of unit cells in the length A. In the same way, n_b_ and n_c_ are, respectively, the number of unit cells in the B and C length [19,20]. Figure 1 shows an example of the decomposition of a bigger block (right-hand side of Figure 1) into a simple lattice cell (left-hand side of Figure 1).

Despite the number of possible geometries for each unit cell, the relative density ρ* is an interesting characterization of a lattice structure.

ρ* = V_L_/V_b_

where V_L_ is the volume of the unit cell with the lattice structure, and V_b_ is the volume of the unit cell if filled with material.

By specific parameters of individual lattice types, it is possible to create a structure with specific mechanical properties with respect to the applied loading. There are some lattice structures (such as body-centered cells) which have the same mechanical properties in all directions. This is applicable for more complex loading scenarios. Some other structures can be, for example, reinforced in the vertical direction (or honeycomb structures), and these structures are suitable for compression. It is possible to consider them as orthotropic.

### 2.1. Two-Dimensional Lattice Structures

**Figure 2:**
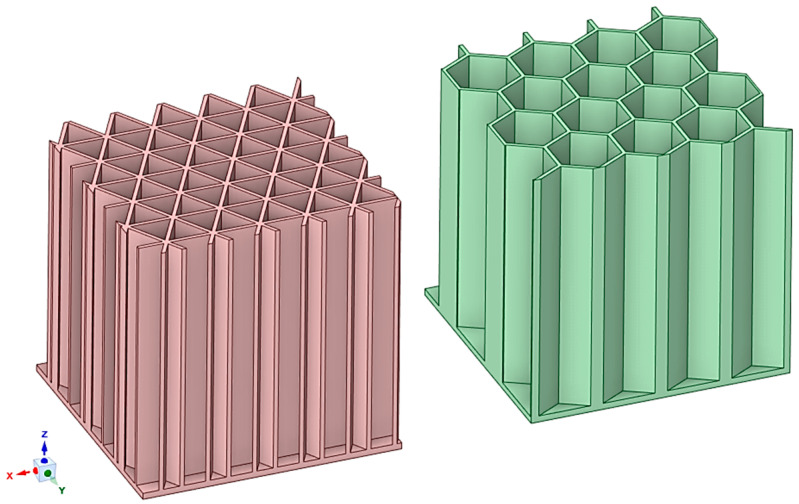
Example of two types of 3D-printed 2D lattice structure with different cross-sections.

Some lattice structures can be two-dimensional (2D), meaning they are composed of 2D unit cells that fill up a given 2D space [21]. These 2D lattice structures can then be extruded in a direction perpendicular to the plane, transforming them into three-dimensional (3D) structures [22,23] (see Figure 2 with 3D-printed honeycomb specimens). Figure 2 shows two different types of honeycomb structures with differences in their extruded cross-section. This change can affect the specific mechanical properties in compression and also in the bending of these structures. This extrusion process can therefore result in a structure with anisotropic mechanical properties, meaning that the material exhibits different mechanical behavior depending on the direction of the applied forces [24,25].

These types of structures can be observed both in nature and in human-made designs. A natural example is the honeycomb, which features a hexagonal pattern that provides exceptional strength and efficiency [26]. The honeycomb structure is renowned for its ability to support weight while using minimal mass, making it an excellent model of natural engineering.

In human-made applications, 2D lattice structures are often utilized for their advantageous mechanical and geometrical properties [27]. One such example is a bottle rack, which uses a grid-like arrangement to hold bottles securely while optimizing space and material usage [28]. The extruded 2D design provides stability and strength, ensuring that the bottles are kept in place while minimizing the amount of material needed for construction.

### 2.2. Periodic Strut-Based Structures

Strut-based structures are characterized by rods or struts that connect nodes in a regular, periodic pattern, effectively filling an entire volume or surface. This type of structure is commonly seen in various large-scale engineering applications due to its inherent strength and stability [29,30].

On a macroscopic scale, strut-based structures form the basis of scaffolding, cranes, and certain types of bridges. In scaffolding, the network of struts provides support and stability for workers and materials during construction or maintenance projects. The periodic arrangement of these struts ensures that the load is evenly distributed, preventing any single point from bearing too much weight and potentially failing [30,31].

**Figure 3:**
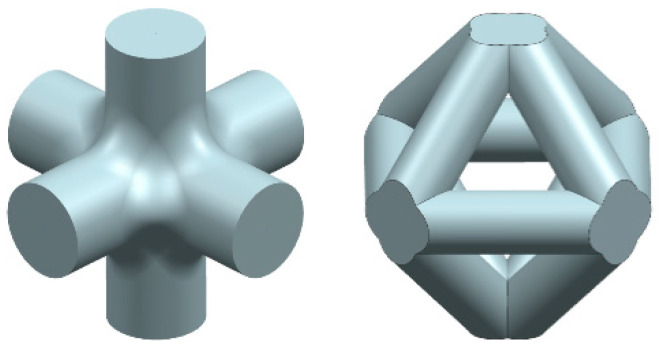
Example of strut-based lattice structures.

Cranes also rely on strut-based lattice structures to achieve the necessary balance of strength and flexibility. The lattice design allows cranes to lift heavy loads while maintaining structural integrity and resisting deformation. The use of lattice-like structures in cranes also diminishes the contribution of the decentered part of the structure in the position of the center of gravity, allowing them to move heavy loads without the whole structure tilting over. Similarly, certain bridge designs, such as truss bridges, use a lattice of struts to distribute loads and provide stability over spans [32,33]. The geometric arrangement of the struts ensures that the bridge can support significant weights while minimizing material usage [34]. These lattice structures (Figure 3) are particularly effective at providing good stiffness and compliance. Stiffness refers to the ability of a structure to resist deformation under load, while compliance is the ability to deform in a controlled manner when subjected to forces. The periodic pattern of struts in these structures helps achieve an optimal balance between these two properties, making them ideal for applications where important parameters such as strength and flexibility are required [35].

## 3. Deformation Modes of Strut-Based Lattice

**Figure 4:**
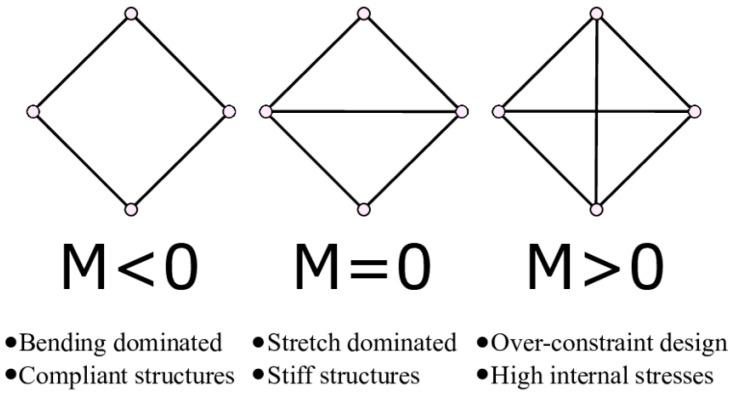
Maxwell’s number definition.

For strut-based lattice structures (either periodic of stochastic), in addition to the lengths and thickness of the struts, a parameter called M, representing the connectivity of the lattice, can help describe its properties (Figure 4) [36]. The local connectivity is the number of struts connected to a node and the global connectivity is the average number of struts per node.

According to Maxwell’s stability criterion M = s − 2j + k, the connectivity of a lattice tells if a structure tends to be stretch dominated or bending dominated [37].

Where

M is Maxwell’s number

S is the number of struts per unit cell

j is the number of joints per unit cell

k is the number of kinetic movements allowed per node [3 in 2D (2 translations and 1 rotation) 6 in 3D (3 translation and 3 rotations)]

## 4. Economic and Ecological Aspects

As presented before, most of the time, AM technologies enable the production of products using only the necessary amount of raw materials, whereas machine manufacturing generates waste. Moreover, the environmental impact caused by AM mainly comes from the energy usage necessary for the printing of the product in question. For machining, on the other hand, a substantial part of the environmental impact comes from material waste [38,39]. The mass reduction generated by the use of lattice structures leads to a reduction of printing time and therefore reduces the amount of energy used for the printing of a single part. Since the cost per part resides mainly in energy consumption, the use of lattice structure reduces both the cost per part and the environmental impact [40].

Moreover, the raw material saved by the volume reduction can sometimes be reused to make other parts, reducing once more the amount of waste and lowering the cost per parts. For some 3D-printing methods, such as MJF or selective laser sintering (SLS), using a powder bed to add layer over layer, the nonmelted powder can most of the time be reused if mixed with new powder, thus avoiding significant waste [41,42].

On the other hand, since 3D printing requires little or no outside intervention, labor costs are eliminated [43,44].

The ecological arguments for using a lattice structure are similar to the economic ones. Indeed, the reduction in volume reduces fuel consumption for transport and, therefore, CO_2_ emissions per piece. Saving nonmelted powder avoids the disposal of potentially polluting waste. And, finally, the reduction of printing time reduces the energy consumption, diminishing the CO_2_ emissions per part [45,46].

In addition to all the economic and ecological benefits, 3D printing also enables the use of materials that subtractive manufacturing cannot. For example, parts can be printed in eco-efficient materials such as compostable biopolymers.

Multi-head printers can also allow the printing of parts composed of multiple materials. They can enable parts with equivalent mechanical properties to be printed using less-polluting materials.

AM technology also enables industries to devote more time to product development, produce small series, and improve products based on customer feedback. This counteracts the current tendency of large chains to fragment demand into several product versions and shorten their life cycles [47,48,49].

**Figure 5:**
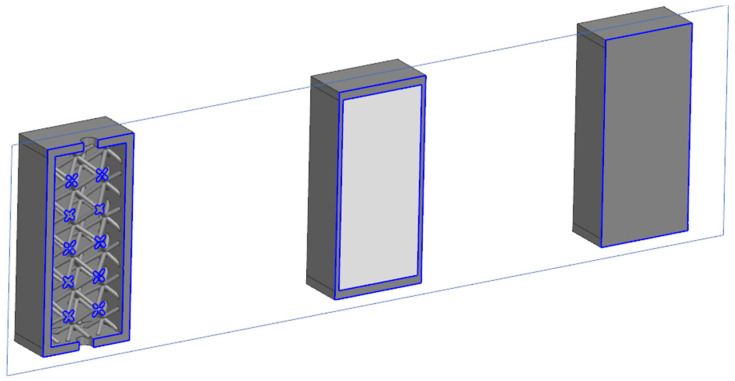
Specimens for cost analysis; lattice with powder and solid (from **left** to **right**).

The economic (price/cost) analysis for parts is shown in Figure 5. Parts are presented in cross-section; the dark gray color represents solid material, and the light gray represents natural powder. The left-hand specimen is with a lattice structure inside but without powder; it is blown through the holes. The middle specimen has a powder inside; it is necessary to take it into account when calculating the price. The right-hand specimen is a completely solid material PA12GB.

The production price for samples can be calculated as follows:

The lattice specimen costs 19.6 EUR. The specimen with powder costs 13 EUR + 2.2 EUR = 15.2 EUR. The costs of powder are incorporated as follows: 1 kg of powder costs 56.5 EUR/1 dm^3^ costs 33 EUR. The volume of powder in the box is 65.3 cm^3^. The price for the volume of powder inside is 2.2 EUR. The solid specimen costs 29 EUR.

It is clear that the cost of a part with the same dimensions can be reduced by 30% using a lattice structure or by 50% using a closed powder. It is necessary to mention that the resulting price is based not only on the volume (smaller total volume results in a lower price) but also on the total area. On the other hand, with fine lattice structures, the area increases, and, thus, the price rises slightly. In the selected case, the weight was reduced to approx. 30% for the lattice sample and approx. 55% for the powder sample.

## 5. Material Models for 3D-Printed Lattice Structures

The production of specimens was performed by usage of MJF technology by the 3D printer HP MJF 4200 (HP, Palo Alto, CA, USA). The source of the material is powder of polyamide with 40% glass. The process was considering the 100% infill of material, and the orientation of samples was 45° with respect to the base plane. The building speed of the print was 4115 cm^3^ per hour.

### 5.1. Isotropic Model

The most straightforward and sensible material model to use for solid PA12GB is a perfectly elastic isotropic material model. This model assumes that the material behaves in a linear elastic manner, where the stress–strain behavior is directly proportional and remains constant regardless of the direction of the load. Such an assumption simplifies the analysis by treating the material as having uniform properties in all directions (isotropic), despite the inherent anisotropy introduced by the 3D-printing process [50,51].

The anisotropy of 3D-printed materials is typically due to variations in mechanical properties along different axes, primarily influenced by the direction of the print layers. However, this study will be neglecting these anisotropic effects (lower than 10%). Ignoring the anisotropic behavior induced by layering simplifies the model, making it more manageable and less computationally demanding [52].

The perfectly elastic isotropic model is computationally efficient because it avoids the complexities associated with nonlinear behavior, anisotropy, and time-dependent properties such as viscoelasticity. This efficiency is particularly advantageous when running simulations on complex structures such as lattice-based structures.

Additionally, the focus of this study does not include the plastic deformation (the material’s permanent deformation after the removal of the load) or damaging of the specimens. Since these factors are not being considered, the simpler, perfectly elastic approach remains viable and appropriate for the purposes of this study.

### 5.2. Anisotropic Model

**Figure 6:**
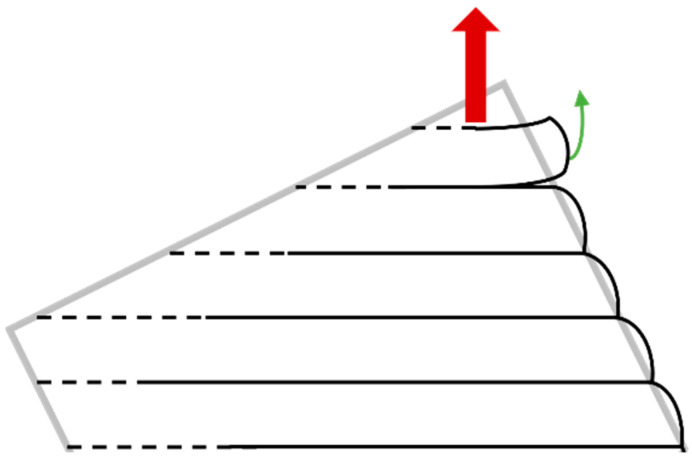
Principle of layers connection.

As explained above, the 3D-printing process inherently induces anisotropy in the specimen’s structure due to its layer-by-layer method of material deposition. These mechanical properties of the printed part can vary depending on the direction of the applied load relative to the orientation of the print layers. Typically, the strength and stiffness of the material are different in the layer deposition direction compared to the directions perpendicular to it, resulting in anisotropic behavior. This is mainly due to the noncontinuity in the perpendicular direction caused by the sticking in-between layers (Figure 6).

This model was not used in the research, although it is the most accurate approximation to reality. It is also beneficial to connect the anisotropic model with the plastic description of behavior, which describes the material correctly during collapsing and rupture. Also, thermal-dependent properties describe the behavior more accurately. Usage of these parameters (anisotropy, plasticity, thermal-dependent properties) can be generally neglected (depending on the purpose of simulation) because they make the simulation process up to 100 times longer compared to usage of the simple isotropic material. Overall, 90% of simulations in product design are in the area of validity of Hooke’s law. Therefore, it is possible to neglect the behavior outside this area and thereby simplify the material model. As shown below, the deviation caused by the simplification (but not only by this) does not exceed 8%, which is acceptable with regard to the generally considered safety in the design.

The specimens are printed at an angle within the printer. This tilted orientation is strategically chosen to minimize the area of each printed layer, thereby reducing the likelihood of printing errors. Printing large surface areas can lead to issues such as warping and shrinking due to uneven cooling and residual stresses. By tilting the specimens, the printing process aims to distribute these stresses more evenly and improve the dimensional accuracy and structural integrity of the printed parts.

**Figure 7:**
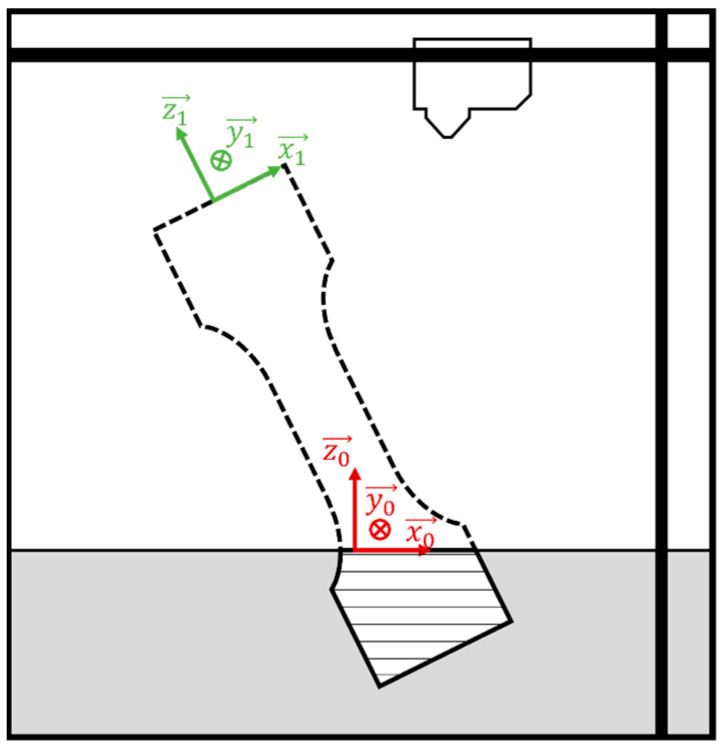
Main direction orientation.

However, this tilted printing direction means that the principal material directions do not align with the principal stress directions when the specimen is subjected to loading (Figure 7). Specifically, the printing direction is not aligned with the neutral axis of the specimen, which is the axis that experiences the least strain during bending. This misalignment complicates the accurate representation of the material’s mechanical behavior. As was mentioned before, the anisotropic material model was not used within this research due to its complexity.

### 5.3. Model of Powder

If the characteristics of the material in terms of stiffness does not have any impact on the response of the structure, the simplest model is a perfectly elastic isotropic material model with a really low Young’s modulus. This model would approximately convey the mass repartition without impacting other types of mechanical characteristics.

To bring the solid model approach for the powder closer to reality, the model should represent the mains characteristics of the powder. It should take into account the powder’s ability to transmit forces in compression and its inability to transmit forces in tension.

**Figure 8:**
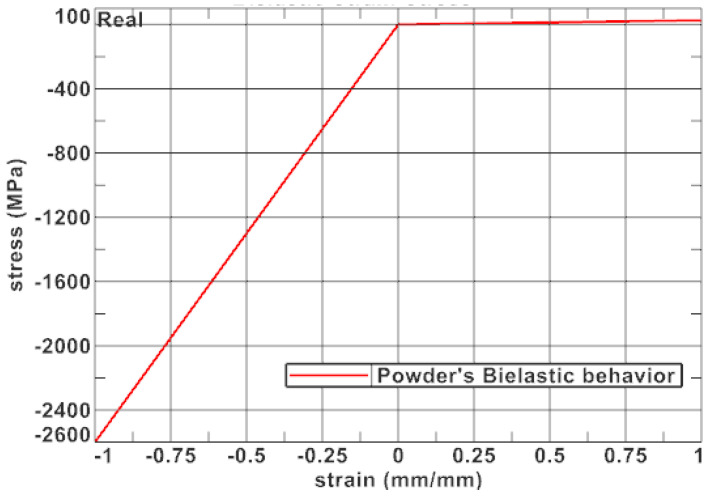
Description of bi-elastic material.

To achieve such characteristics, a good approach could be a bi-elastic material (Figure 8) with a bigger Young’s modulus in compression and a negligeable one in tension. This is a rough approach that does not represent the powder’s capacity to densify itself under compression but which is easy to be numerically modeled.

## 6. Compression Test

**Figure 9:**
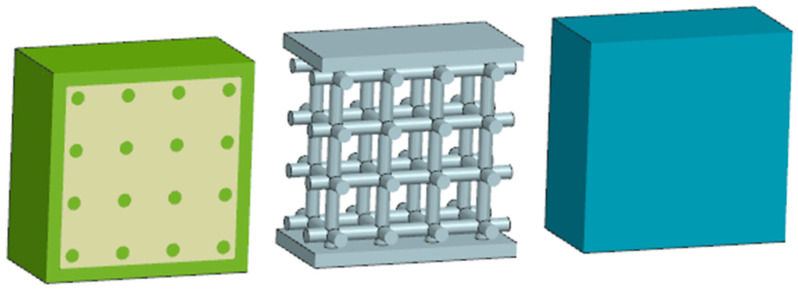
Compression specimens.

The testing was performed on 3D-printed specimens according the following description: The solid specimen (marked “S”, blue color in Figure 8) is a 30 mm PA12GB cube. All other geometries are derived from this solid. The lattice specimen (marked “L”, grey color in Figure 9) has 2 mm diameter struts, and the boxed lattice with powder (marked “BLP”, green color in Figure 8) is filled with PA12GB powder. For the compression tests, the geometries used for the experimentation and the simulations were simple bricks of PA12GB with a side length of 30 mm. The parameters during testing were a speed of 10 mm per minute, constant loading, and an ambient temperature in the testing room (22 °C). The hydromechanical testing machine was used for this test.

### Data Processing

**Figure 10:**
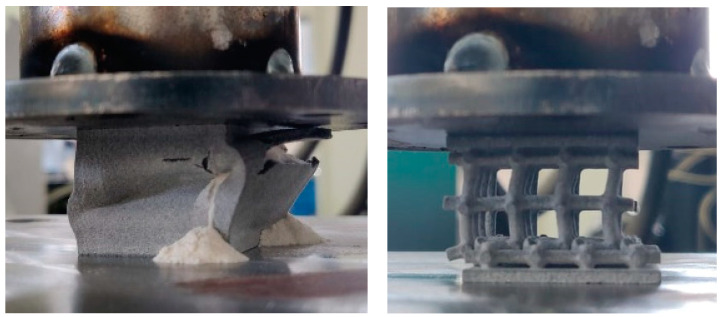
Two types of specimens during testing; “boxed lattice with powder” (**left**) and “lattice specimen” (**right**).

At the beginning of each force–displacement curve, a small, nonlinear portion is observed. This initial nonlinearity can be explained by the mechanical influence of gaps between the components of the press, which need to be taken up before the full load is applied to the specimen. Once these gaps are closed, the force is transmitted more directly to the specimen, resulting in a linear response that reflects the material’s elastic behavior. It should also be noted that the reaction force also takes into account the stiffness of the machine, which is not determined in this study. An example of the testing is shown in Figure 10. It is obvious that for the boxed lattice specimen with powder, the collapse of the side wall is the crucial point. After this point, the structure loses its stability, and the stiffness is reduced significantly. For the lattice structure without powder, the behavior of the collapsing is interesting. It is obvious that layers (oriented perpendicularly to the compression force) are collapsing gradually, one by one. Each collapse of an individual layer is connected with the individual peak of stiffness. From an operational point of view, the course of behavior until the collapse of the first layer is important.

**Figure 11:**
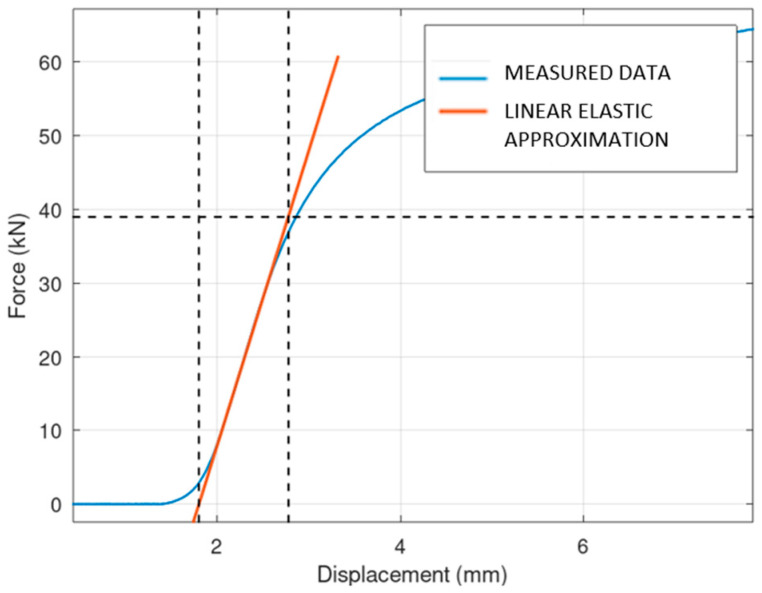
Linearization of solid specimen.

**Figure 12:**
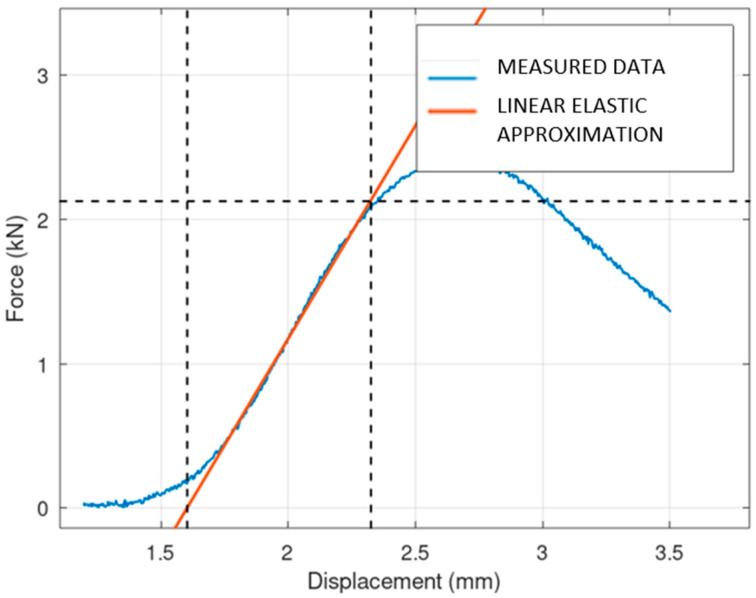
Linearization of lattice specimen.

**Figure 13:**
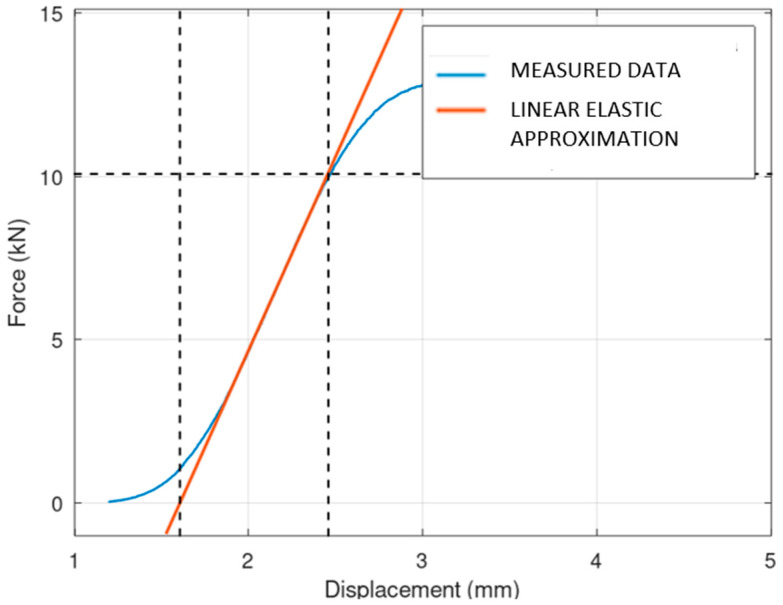
Linearization of boxed lattice specimen with powder.

The results of mechanical compression testing for all types of specimens are shown in the following three figures (Figure 11, Figure 12 and Figure 13). The dependency between the applied force and displacement is shown. The testing results are in blue, and the linear elastic approximation of the results is shown in red. It is obvious that the solid specimen is able to survive the highest loading, and the lattice the lowest loading.

An interesting fact is the unsmoothed course of the measurement for the lattice sample, which corresponds to the gradual breaking of the individual layers.

For this simulation, the specimens’ displacement was constrained in the Z direction in order to simulate the plan support. Some points of the specimens were also chosen to be constrained in the x and y directions to avoid any eventual solid body movements. Since the loading was slow, an eventual viscoelastic behavior can be neglected, therefore a static analysis using an enforced displacement boundary conditions on the upper plan is sufficient to describe the action of the hydraulic press on the specimens.

**Figure 14:**
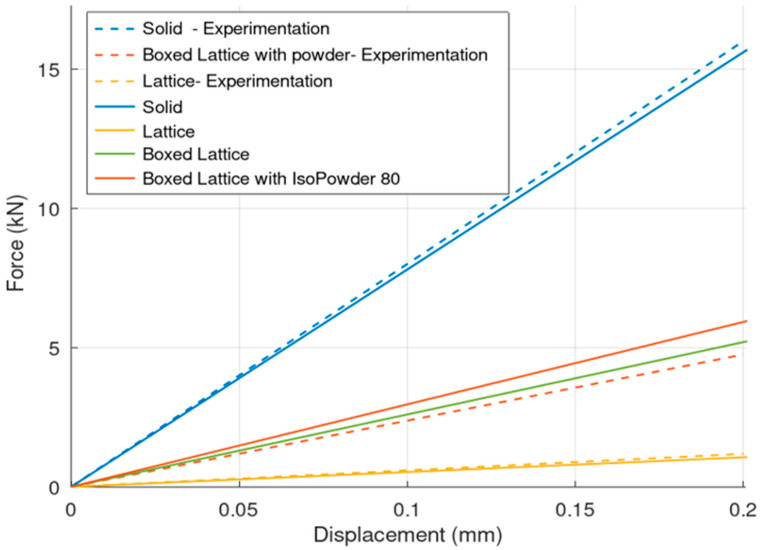
Comparing of simulation and testing.

Comparing the simulation of the S, L, and BLP specimens with the experimental results gives more insight into the behavior of the powder (Figure 14). It is obvious that the material description is sufficient.

## 7. Conclusions

This study was focused on the elastic behavior of three structures: S (blue), BLP (red), and L (yellow). In Figure 13, the dashed lines represent interpolated experimental data, while the solid lines show simulation results.

The experimental findings reveal that the simulation error is less than 8%, with potential sources of error linked to measurement inaccuracies. One notable source of error is the inherent stiffness of the testing machine itself. The hydraulic press used in the experiments possesses its own stiffness, which can influence overall measurements. When force is applied to the specimen, some deformation occurs within the machine’s components rather than solely in the specimen. This additional machine stiffness is not considered in the simulation, which assumes an ideal setup where deformation is confined to the specimen alone. another obvious source of the different behavior between experiment and simulation is the simplification of the material models to be purely isotropic.

Interestingly, the difference between the simulated and interpolated results for the BLP (powder-filled) structures is minimal. This suggests that the presence of powder within the BLP specimen does not significantly affect the structure’s stiffness during compression. This research does not describe the effect of this powder for different loading scenarios (for example, during vibrations, where this powder will have a significant damping effect). This inference is supported by observations during compression tests where the powder slowly flowed out as the walls of the BLP specimen breached. If the powder had been tightly constrained within the cavity, the internal pressure would have been much higher, resisting the caving of the walls. In such a scenario, tightly packed powder would likely have burst out forcefully as soon as the specimen’s walls breached. However, this was not observed.

The slow flow of powder indicates that it is not significantly constrained and does not substantially contribute to the structure’s overall stiffness. In a highly constrained environment, powder density would increase as the grains compacted, eventually behaving more like a solid and providing additional resistance to deformation. However, in this case, the powder moved relatively freely within the cavity, offering minimal resistance and having little impact on structural stiffness.

The powder has a density of 0.48 g/cm^3^ compared to 1.30 g/cm^3^ for the solid material, meaning 63% of the powder’s volume consists of air. Since the nonhollow specimens enclose the powder, there is also about 63% air in the cavity. Under constraints, the powder grains can rearrange and densify, potentially behaving like a solid material (similar to the principle of sintering). But without sufficient constraint, the powder retains its “powder-like” behavior.

This analysis suggests that, under compression, the powder-filled structure behaves similarly to a hollow structure in terms of stiffness. The minimal difference between simulated and experimental results supports the conclusion that the powder does not play a significant role in altering stiffness. This finding is crucial for understanding the mechanical behavior of such composite structures and can inform future design and material choices.

While the powder does not affect the compressive behavior of the specimens, its mass cannot be ignored. Future research will explore different specimen configurations and materials, focusing on different loading conditions, particularly vibration and fatigue, where the powder’s effect may be more pronounced. The same procedure will be applied to materials without glass filling, particularly the more elastic AM-produced polymers.

## Data Availability

The original contributions presented in the study are included in the article, further inquiries can be directed to the corresponding author.
